# Nephrotic syndrome with acute kidney injury due to combination therapy of immune checkpoint inhibitors: a case report and review of the literature

**DOI:** 10.1186/s12882-024-03494-1

**Published:** 2024-02-09

**Authors:** Ryosuke Saiki, Kan Katayama, Haruko Saiki, Ayumi Fukumori, Kayo Tsujimoto, Masahiro Yamawaki, Fumika Tanaka, Daisuke Takahashi, Keiko Oda, Yasuo Suzuki, Tomohiro Murata, Kaoru Dohi

**Affiliations:** 1 Department of Cardiology and Nephrology, Mie University Graduate School of Medicine, 2-174 Edobashi, Tsu, Mie 514-8507 Japan; 2 Department of Pulmonary and Critical Care Medicine, Faculty and Graduate School of Medicine, Mie University, Tsu, Japan

**Keywords:** Nivolumab– ipilimumab, Immune checkpoint inhibitors, Nephrotic syndrome, Acute kidney injury, Minimal change disease

## Abstract

**Background:**

Recent studies have focused on immune checkpoint inhibitors. Renal complications associated with the use of immune checkpoint inhibitors are uncommon compared with other immune-related adverse events. Acute interstitial nephritis accounts for most of these renal complications, with nephrotic syndrome quite rare. We herein report a case of nephrotic syndrome associated with immune checkpoint inhibitors that was more severe than that in previous cases. By comparing this case with previous reports, the possible reasons for the particular severity of this case are discussed.

**Case presentation:**

A 75-year-old man developed nephrotic syndrome with acute kidney injury after the first combination therapy of nivolumab and ipilimumab for malignant pleural mesothelioma. The results of a kidney biopsy indicated minimal change disease with mild atherosclerosis, acute interstitial nephritis, and fusion of nearly all podocyte foot processes. Nivolumab and ipilimumab therapy were stopped, and treatment with corticosteroids was initiated. We investigated previously reported cases of nephrotic syndrome using immune checkpoint inhibitors. Seventeen cases of immune checkpoint inhibitor-related nephrotic syndrome, including ours, have been reported. Two of the 17 patients with immune checkpoint inhibitor-related nephrotic syndrome required hemodialysis treatment for acute kidney injury. Unlike many previously reported cases, the present patient was administered two different immune checkpoint inhibitors, which may be one of the reasons for the development of severe nephrotic syndrome.

**Conclusions:**

In addition to previously reported risk factors, immune checkpoint inhibitor combination therapy can exacerbate nephrotic syndrome compared to immune checkpoint inhibitor monotherapy.

## Background

Recent studies have focused on immune checkpoint inhibitors (ICIs) [1]. In patients with unresectable tumors, ICIs are used as the first-line therapy in a variety of fields [2, 3], which has contributed to substantially improved survival [2]. For example, malignant pleural mesothelioma is a highly aggressive malignancy that is often unresectable at time of the diagnosis, with less than 10% of patients surviving five years or longer [4]. Nivolumab plus ipilimumab have provided significant and clinically meaningful improvements in overall survival versus standard-of-care chemotherapy, which supported its approval in the USA as the first-in-class regimen for previously untreated unresectable malignant pleural mesothelioma [5].

Patients treated with ICIs often develop immune-related adverse events (irAEs), which are immune-mediated secondary effects of ICIs [1].

Renal complications associated with the use of ICIs are uncommon compared with other irAEs [6]. Most cases of renal complications are acute interstitial nephritis, which is said to account for 93% of cases of ICI-related acute kidney injury [7]. In contrast, nephrotic syndrome is extremely rare, as is minimal change disease [2, 6, 8–11].

We herein report a case of nephrotic syndrome associated with ICIs that was more severe than that in previous cases. By comparing this case with previous reports, the possible reasons for the particular severity of this case are discussed.

## Case presentation

A 75-year-old man received first-line nivolumab (treatment dose, 360 mg every 3 weeks) plus ipilimumab (1 mg/kg intravenously every 6 weeks), combination therapy with anti-programmed cell death 1 (anti-PD-1) antibody, and cytotoxic T-lymphocyte-associated antigen 4 (CTLA-4) antibody for malignant pleural mesothelioma. His comorbidities included an artificial anus due to perforation of the sigmoid colon and slight proteinuria (urine dipstick test: 30 mg/dl). He did not have any red blood cells, white blood cells, or casts in his urine. His serum albumin, serum creatinine, and hemoglobin A1c values were 3.7 g/dl, 0.82 mg/dl, and 6.0%. He had a history of smoking from 20 to 60 years old. He did not normally take regular medication, but had been taking loxoprofen sodium (60 mg, three times daily), tramadol hydrochloride (37.5 mg, four times daily), and acetaminophen (325 mg, four times daily) for two months for pain caused by the tumor. After the first dose of ICIs (13 days after the initial treatment), he suddenly presented with leg edema, massive proteinuria (19.85 g/day), urinary glucose (0.344 g/day), and hypoalbuminemia (2.0 g/dl). His blood pressure at presentation was 97/48 mmHg. Based on these results, the patient was diagnosed with nephrotic syndrome.

**Fig. 1:**
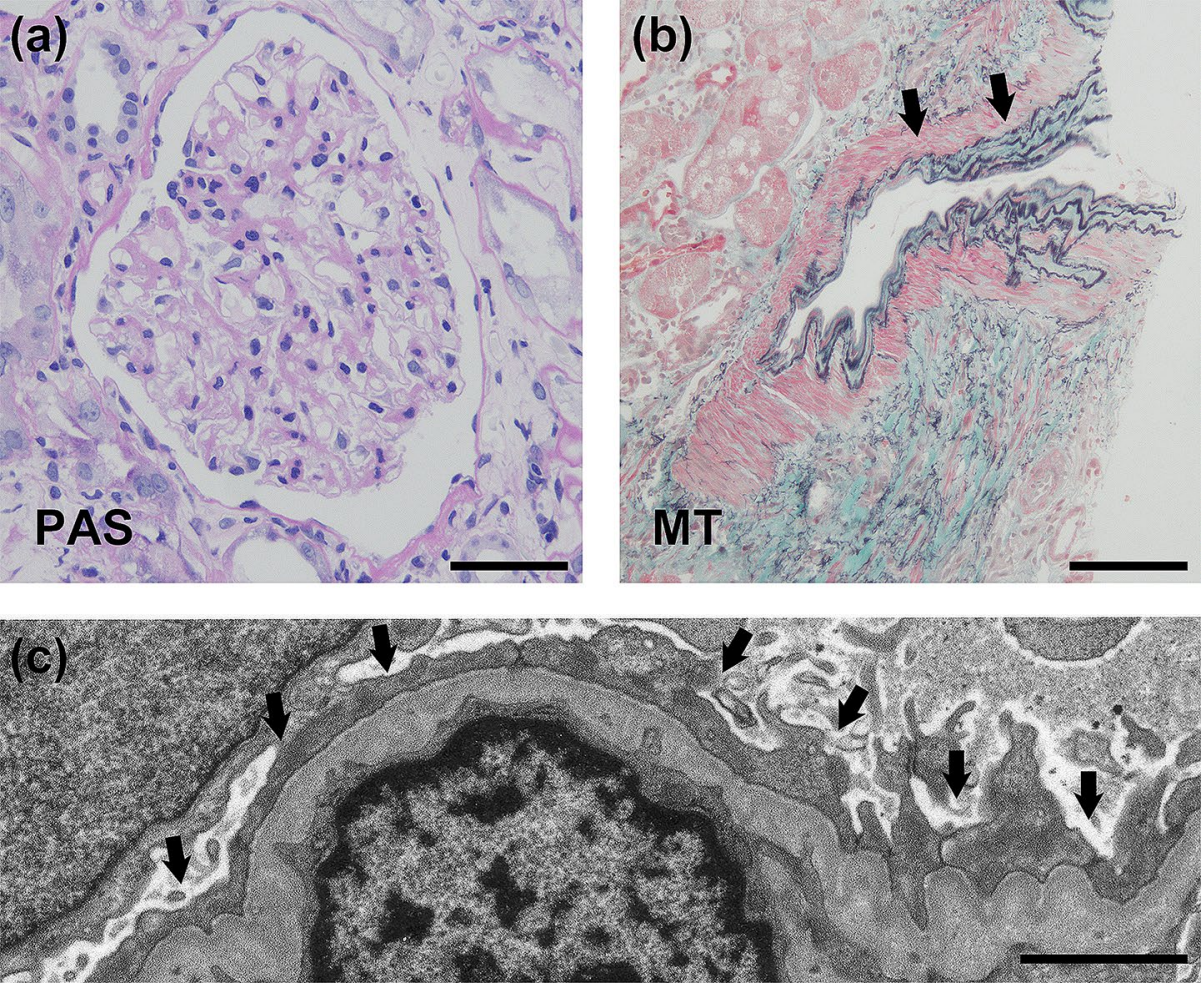
Kidney biopsy findings. (**a**) Periodic acid Schiff (PAS) staining did not show focal sclerosis or increase in mesangial matrix or cellularity. Bar = 50 μm. (**b**) Masson-Trichrome (MT) staining showed medial hypertrophy and fibroblastic intimal thickening (arrows) of the arch arteries. Bar = 100 μm. (**c**) Representative electron micrograph obtained from a kidney biopsy. Extensive podocyte foot process fusion was revealed (arrows), without any electron-dense deposits. Bar = 2 μm

The administration of loxoprofen sodium was discontinued, and a kidney biopsy was performed, and light microscopy revealed 11 glomeruli without focal sclerosis, an increase in the mesangial matrix, or cellularity (Fig. 1a). The renal interstitium exhibited mild fibrosis (Fig. 1b), mild leukocytic infiltration, and slight tubulitis, accompanied by medial hypertrophy and fibroblastic intimal thickening in the interlobular and arcuate arteries. An immunofluorescence analysis yielded negative results. Electron microscopy revealed fusion of nearly all podocyte foot processes without any electron-dense deposits (Fig. 1c). Overall, the biopsy specimens indicated minimal change disease (MCD) with mild atherosclerosis and acute interstitial nephritis (AIN).

Nivolumab and ipilimumab therapy were stopped, and treatment with corticosteroids (prednisone, 1 mg/kg) was initiated. However, diuretic medicines did not work well, resulting in the need for hemodialysis initiation, and excess fluid was removed. His urinary protein and glucose levels gradually decreased with continued treatment and prednisone was tapered. Hemodialysis was discontinued because his urine output gradually increased 35 days after initiating hemodialysis.

**Fig. 2:**
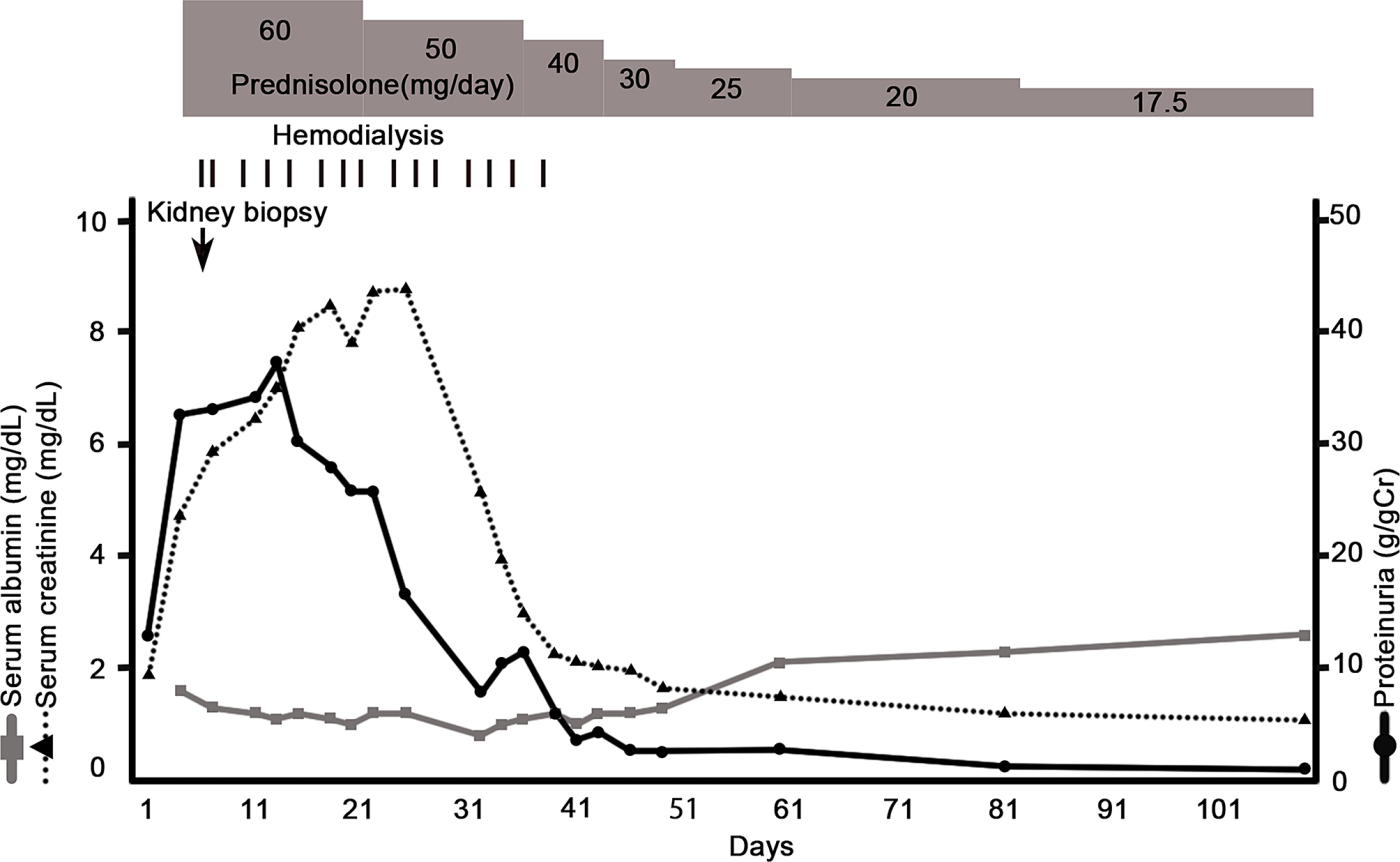
The clinical course

Since discharge, his serum creatinine and urine albumin-to-creatinine ratio was 1.06 mg/dl and 1.06 g/gCr. The patient has continued to attend outpatient visits in good spirits, and prednisone tapering is ongoing (Fig. 2). Regarding the malignant pleural mesothelioma, the patient will be monitored to see if it grows, without re-administration of ICIs. If it grows, other chemotherapy will be administered.

## Discussion and conclusions

We encountered a case of nephrotic syndrome with MCD caused by an ICI. One proposed mechanism concerning how ICIs lead to podocyte injury is remote production of a “permeability factor” that may cause release of cytokines that then promote podocyte foot-process effacement [8]. Although MCD secondary to cancer is well-recognized, there is evidence that the present case was related to therapy rather than to the malignancy itself. First, nephrotic syndrome developed soon after administration of the first ICIs in this case. In previous reports, approximately 30% of ICI-related acute kidney injury occurred within 5 weeks, with a median time from administration to the onset of 14 weeks [7]. Second, in cases of MCD secondary to cancer, the course of proteinuria typically parallels that of the underlying malignancy, with MCD remitting after successful cancer treatment [12]. Computed tomography, performed when the patient had severe renal failure requiring dialysis, showed that his tumor had shrunk dramatically. These findings indicated that MCD is caused by ICIs. Furthermore, some studies showed an association between the occurrence of irAEs and the survival in patients treated with ICIs [1, 13, 14], which may explain why the present case with severe nephrotic syndrome showed dramatic tumor shrinkage. In addition to MCD, this patient also presented with AIN, albeit in a mild form. Loxoprofen sodium, similar to ICIs, is known to potentially induce MCD and AIN. Although the risk of non-steroidal anti-inflammatory drugs (NSAIDs) related nephrotic syndrome persists for up to 2 years after discontinuation of NSAIDs, it is particularly high within the first 2 weeks of exposure [15]. The patient had been taking loxoprofen sodium for 2 months prior to the onset of nephrotic syndrome. The diagnosis of nephrotic syndrome was attributed to ICIs, considering the timeline between drug administration and the onset of the disease.

We investigated previously reported cases of nephrotic syndrome using ICIs. PubMed was searched using the following two formulas: (1) (“Antibodies, Monoclonal, Humanized”[MeSH Terms] OR “immune checkpoint inhibitors”[MeSH Terms]) AND (“Nephritis”[MeSH Terms] OR “Nephrosis”[MeSH Terms]), and (2) (PD-1 OR CTLA-4 OR “immune checkpoint inhibitors”) AND (“minimal change disease” OR nephritis); 444 and 424 articles, respectively, were identified using the search strategies. We checked all titles and abstracts of the articles to identify pertinent articles, and then examined the contents of the remaining papers to determine their applicability to this investigation.

Our survey identified 17 cases of ICI-related nephrotic syndrome, including our own (Table 1) [8–11, 16–24]. The ratio of males (14) to females (3) was 4.7:1. The patients’ age ranged from 40 to 75 years, with an average age of 62.1 years old. Lung adenocarcinoma was the underlying condition in five cases, malignant melanoma in five, malignant pleural mesothelioma in two, renal cell carcinoma in two, Hodgkin lymphoma in two, and chondroma in one. Pembrolizumab was the most commonly used ICI, with multiple ICIs used in two cases. The pathological types of nephrotic syndrome were as follows: MCD in 7 (41.1%), membranous nephropathy in 3 (17.6%), focal segmental glomerulosclerosis in 2 (11.8%), IgA nephropathy in 1 (5.9%), AA-type amyloidosis in 1 (5.9%), pauci-immune glomerulonephritis with crescents in 1 (5.9%), and not performed in 1 (5.9%). Most cases developed nephrotic syndrome within a few months of initial treatment, but some occurred more than a year later. As the first treatment, most patients were administered high-dose prednisone (approximately 1 mg/day), and in 1 case [16], only the ICI was discontinued. The patient was observed; however, there was no improvement, and prednisone was eventually initiated. Most patients did not require dialysis, with dialysis being necessary in only 2 cases (11.8%).

**Table 1:** Summary of reported cases of ICI-related nephrotic syndrome

Authors	Age, sex	Underlying malignancies	Anti-PD-1	Number of doses at onset	Renal pathological diagnosis	Creatinine (mg/dl)	Alb (g/dl)	Proteinuria (g/day)	Proteinuria (g/gCr)	Initial steroid dose	Dialysis
Fadel, et al. 2009	60, M	Malignant melanoma	Ipilimumab	2	Lupus nephritis	1.01	2.45	7.5	NA	1 mg/kg/day	None
Bickel et al. 2016	62, M	Malignant pleural mesothelioma	Pembrolizumab	1(10 days from initial treatment)	MCD	NA	1.5	19	NA	1 mg/kg/day	None
Daanen et al. 2017	62, M	RCC	Nivolumab	4(7–8 weeks from initial treatment)	FSGS	2.95	1.9	17	NA	pulse 3days -> 60 mg/day	None
Kitchlu, et al. 2017 -1	43, M	Hodgkin lymphoma	Pembrolizumab	2(4 weeks from initial treatment)	MCD with acute tubular injury	3.93	1.8	10.3	NA	2 mg/kg/day	None
Kitchlu, et al. 2017 -2	45, M	Malignant melanoma	Ipilimumab	4(18 months into the treatment course)	MCD	0.8	2.6	9.5	NA	1 mg/kg/day	None
Gao, et al. 2018	40, M	Hodgkin lymphoma	Camrelizumab	3(65 days from initial treatment)	MCD	0.9	2.1	30	NA	1 mg/kg/day	None
Mamlouk, et al. 2019-1	69, M	Malignant melanoma	Ipilimumab + Nivolumab	2(6 weeks from initial treatment)	IgA nephropathy	2.4	NA	NA	7.7	0.5 mg/kg/day	None
Mamlouk, et al. 2019-2	60, F	RCC	Nivolumab	6(16 weeks from initial treatment)	MN without PLA2R	NA	NA	NA	9.7	1 mg/kg/day	None
Mamlouk, et al. 2019-3	63, M	Chondroma	Pembrolizumab	6(18 weeks from initial treatment)	AA amyloidosis	2.25	NA	NA	31	1 mg/kg/day	None
Glutsch, et al. 2019	68, M	Malignant melanoma	Pembrolizumab	1(18 days from initial treatment)	MCD with acute tubular injury	2.86	1.6	NA	NA	100 mg/day	None
Gallan, et al. 2019	71, F	Lung adenocarcinoma	Pembrolizumab	10 months from initial treatment	Pauci-immune glomerulonephritis with crescents	0.8	NA	5.5	NA	NA	None
Saito, et al. 2020	79, M	Lung adenocarcinoma	Pembrolizumab	7(19-21weeks from initial treatment)	MCD	NA	1.1	NA	13.82	40 mg/day	None
Ishibuchi, et al. 2020	70, M	Lung adenocarcinoma	Pembrolizumab	5 months from initial treatment	NA	1.63	1	NA	15.1	1.6 mg/kg/day	Needed
Chen, et al. 2021	74, M	Lung adenocarcinoma	Tislelizumab	15(11 months from initial treatment)	MN(THSD7A positive)	0.93	1.9	20.16	NA	60 mg/day	None
Kim, et al. 2021	46, F	Malignant melanoma	Pembrolizumab	16 months from initial treatment	FSGS	0.66	2.84	NA	3.277	None	None
Wakabayashi, et al. 2021	69, M	Lung adenocarcinoma	Nivolumab	5 months from initial treatment	MN	1.02	2.2	NA	13.3	0.8 mg/kg/day	None
The present case	75, M	Malignant pleural mesothelioma	Ipilimumab + Nivolumab	1(14 days from initial treatment)	MCD with AIN	1.87	2	19.85	NA	1 mg/kg/day	Needed

AIN, acute interstitial nephritis; Alb, albumin; Cre, creatinine; F, female; FSGS, focal segmental glomerulosclerosis; ICI, immune checkpoint inhibitor; IgA, immunoglobulin A; M, male; MCD, minimal change disease; MN, membranous nephropathy; NA, not available; PLA2R, phospholipase A2 receptor; RRC, renal cell carcinoma; THSD7A, thrombospondin type-1 domain-containing

The present patient had fusion of nearly all podocyte foot processes and a more severe condition than previous cases, and required dialysis. The main factors leading to nephrotic syndrome with acute kidney injury in the general population include but are not limited to old age, hypertension, arterial/arteriolar lesions, and a serum albumin level < 2.0 g/dl [25]. Old age and arterial/arteriolar lesions were present in the present case. He had proteinuria at baseline because of nephrosclerotic changes due to arterial/arteriolar lesions. The urinary protein in this case reflected the risk of acute kidney injury. In addition, this case showed renal glycosuria, indicating complication with tubular dysfunction [26]. Although the pathogenesis of tubular dysfunction in nephrotic syndrome remains unclear, it is hypothesized that increased tubular protein reabsorption leads to a massive influx of protein into the tubular lumen, which in turn directly damages tubular epithelial cells [26]. The detection of tubular dysfunction coincides with worsening of the renal function and more proteinuria than in cases without tubular dysfunction [26]. AIN may also have been involved in acute kidney injury in this case. In this sense, the present case was a severe one. Furthermore, unlike many previously reported cases, our case was administered two different ICIs, which can cause nephrotic syndrome to become severe; the fact that combination therapy induces multiple concurrent irAEs more often than monotherapy [27] supports this hypothesis.

In summary, we encountered a case in which combination ICI therapy (anti-PD-1 and CTLA-4 antibodies) led to severe nephrotic syndrome with acute kidney injury. In addition to previously reported risk factors, such as advanced age and atherosclerosis, ICI combination therapy can increase the risk of nephrotic syndrome compared to ICI monotherapy.

## Data Availability

The datasets used and/or analyzed during the current study are available from the corresponding author upon reasonable request.

## Abbreviations

AIN: acute interstitial nephritis
anti-PD-1: anti-programmed cell death 1
CTLA-4: cytotoxic T-lymphocyte-associated antigen 4
ICIs: immune checkpoint inhibitor
irAE: immune-related adverse event
MCD: minimal change disease
non-steroidal anti-inflammatory drugs: NSAIDs
